# M2 macrophages are more resistant than M1 macrophages following radiation therapy in the context of glioblastoma

**DOI:** 10.18632/oncotarget.19994

**Published:** 2017-08-07

**Authors:** Marine M. Leblond, Elodie A. Pérès, Charly Helaine, Aurélie N. Gérault, Damien Moulin, Clément Anfray, Didier Divoux, Edwige Petit, Myriam Bernaudin, Samuel Valable

**Affiliations:** ^1^ Normandie Université, UNICAEN, CEA, CNRS, ISTCT/CERVOxy Group, 14000 Caen, France

**Keywords:** macrophages, irradiation, glioblastoma, radioresistance, phenotypic selection

## Abstract

In some highly inflammatory tumors, such as glioblastoma (GB), macrophages (MΦ) represent the most abundant population of reactive cells. MΦ, initially denoted as M0 MΦ, can be polarized into two further phenotypes: the antitumor M1 MΦ, and the protumor M2 MΦ. The three phenotypes can reside simultaneously in the tumor mass and various external factors may influence MΦ polarization. Radiotherapy is a common modality of cancer treatment aiming to target tumor cells. However, the specific effects of X-ray radiation on the inflammatory cells are, so far, controversial and not fully understood. In the present investigation, we have first analyzed, *in vivo*, the effect of X-ray radiation on MΦ present in GB tumors. We have observed a decrease in MΦ number paralleled by an increase in the proportion of M2 MΦ. To understand this phenomenon, we then evaluated, *in vitro*, the effects of X-rays on the MΦ phenotypes and survival. We have found that X-ray radiation failed to modify the phenotype of the different MΦ. However, M1 MΦ were more sensitive to ionizing radiation than M2 MΦ, both in normoxia and in hypoxia, which could explain the *in vivo* observations. To conclude, M2 MΦ are more radioresistant than M0 and M1 MΦ and the present study allows us to propose that X-ray radiotherapy could contribute, along with other phenomena, to the increased density in the protumor M2 MΦ in GB.

## INTRODUCTION

Glioblastoma (GB) are the most frequent and aggressive form of the primary malignant brain tumors in adults [1]. Conventional therapy consists of surgery associated with X-ray radiotherapy (5×2Gy per week for 6 weeks, for a total dose of 60Gy) with concomitant and adjuvant chemotherapy based on temozolomide [2]. Despite this therapeutic arsenal, recurrence inevitably occurs and the median survival of GB patients remains around 15 months [3].

GB are highly heterogeneous tumors in which various cell types coexist, such as tumor cells, endothelial cells, fibroblasts and different cell types of the immune system [4]. Of all the different cell types colonizing GB, macrophages (MΦ) are the most abundant infiltrating immune cells and are named tumor associated macrophages (TAM) [5]. Circulating monocytes can migrate towards the tumor and once in the tissue, monocytes differentiate into MΦ, called M0 MΦ, under the influence of cytokines [6]. In GB, the myeloid population is the major player of the innate immune system and represents up to 30% of the tumor mass [7]. Interestingly, the number of MΦ retrieved in the tumor is inversely correlated to the overall survival of GB patients [8].

Once differentiated, M0 MΦ exhibit a considerable degree of plasticity and can be polarized into two well established functional phenotypes, termed M1 and M2 MΦ [9]. M1 MΦ, characterized in part by the expression of the inducible type of nitric oxide synthase (iNOS), are classically activated MΦ implicated in an antitumor activity, exemplified by their phagocytic properties and their capacity to activate the synthesis of pro-inflammatory cytokines [10]. In contradistinction, M2 MΦ are essentially characterized by a potent arginase-1 (Arg1) activity and the CD206 marker. These activated MΦ are known to promote tumor development by tissue remodeling, cell proliferation, immunoregulation and angiogenesis [11]. As we reported recently, within the tumor mass, the three MΦ phenotypes are observed with the predominance of the M0 and M1 phenotypes in the most oxygenated area of the tumor whereas the M2 MΦ are found in the hypoxic/necrotic areas [12]. Beyond its involvement in tumor growth, the MΦ phenotype is also suspected to be associated with a poor response to GB treatments [8].

Ionizing radiations (IR), such as X- and γ-radiations, can also influence the tropism of MΦ in the tumor by an increased production of chemokines at the origin of MΦ migration. In support of the above, studies have demonstrated that irradiation promotes the recruitment of MΦ in brain tumors approximately 20 days post-radiation [13] by increasing the stromal cell derived factor-1 (SDF-1) production [13, 14].

With respect to the phenotype of MΦ following exposure to IR, an increase in M2 markers has been observed, *in vivo*, in various tumor types [13, 15, 16], including GB [17]. Others studies have reported that radiation therapy can also increase M1 markers [18] while others failed to observe any change in cytokine production [19]. X-ray exposure also induces a local reoxygenation [20] which could in turn modulate the MΦ phenotype [12].

Due to the controversial findings in the literature, it is necessary to clarify the MΦ response to irradiation. As of present, the effects of IR exposure on the three MΦ phenotypes have never been analyzed *in vitro* and require investigations not only on the polarization of MΦ but also on their fate.

A better understanding of the effects of X-rays on MΦ phenotype is essential to tailor therapeutic approaches since attention has recently been focused on the role of TAM in the mechanisms of resistance to treatment [21]. Two main hypotheses may be advanced to elucidate as to whether irradiation influences the proportion of one phenotype compared to the others: i) X-ray exposure polarizes or re-educates MΦ; or ii) one phenotype is more sensitive to radiation-induced cell death than the others. To test these hypotheses, we have first evaluated, *in vivo*, the effect of IR on TAM present in GB. In a second stage, we have investigated, *in vitro*, whether X-ray radiation induces a change in the phenotype of M0, M1 and M2 MΦ or whether X-ray radiation is deleterious in the three different phenotypes. Cells were irradiated with either 2Gy or 8Gy of X-rays. 2Gy per day is a dose usually delivered in GB patients. However, as 2Gy produces only modest cell death, 8Gy was also delivered to amplify any differences in the radiosensitivities of the different cell populations. Given the hypoxic nature of GB and that hypoxia represents a factor of poor prognosis and also influences inflammation [12], experiments were conducted under normoxic, moderate (1% O_2_, the O_2_ level commonly found in GB) [22] and severe (0.2% O_2_, the O_2_ level found around necrotic areas of GB) hypoxic conditions, to recapitulate the various oxygen level that are present in the tumor situation.

## RESULTS

### X-ray radiation induces a marked decrease in MΦ number but an increase in M2 MΦ proportion in GB

**Figure 1:**
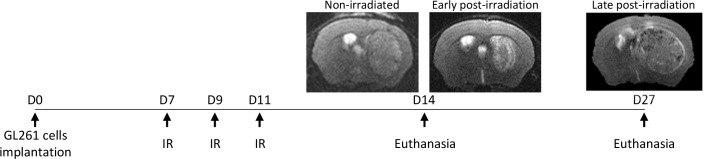
Experimental protocol of the GL261 glioma model with the representative MRI of the non-irradiated, early post-irradiation and late post-irradiation animals Non-irradiated and early post-irradiation tumors were both arrested 14 days after the GL261 cells implantation (non-irradiated tumor volume≈40mm^3^; early post-irradiation tumor volume≈10mm^3^) to match in time and just before the complete regression of the irradiated tumors. Late post-irradiation tumors, corresponding to recurrence, were arrested when the tumor reached 50 mm^3^. This time point does not have non-irradiated tumors because the control tumors developed too rapidly without any treatments.

**Figure 2:**
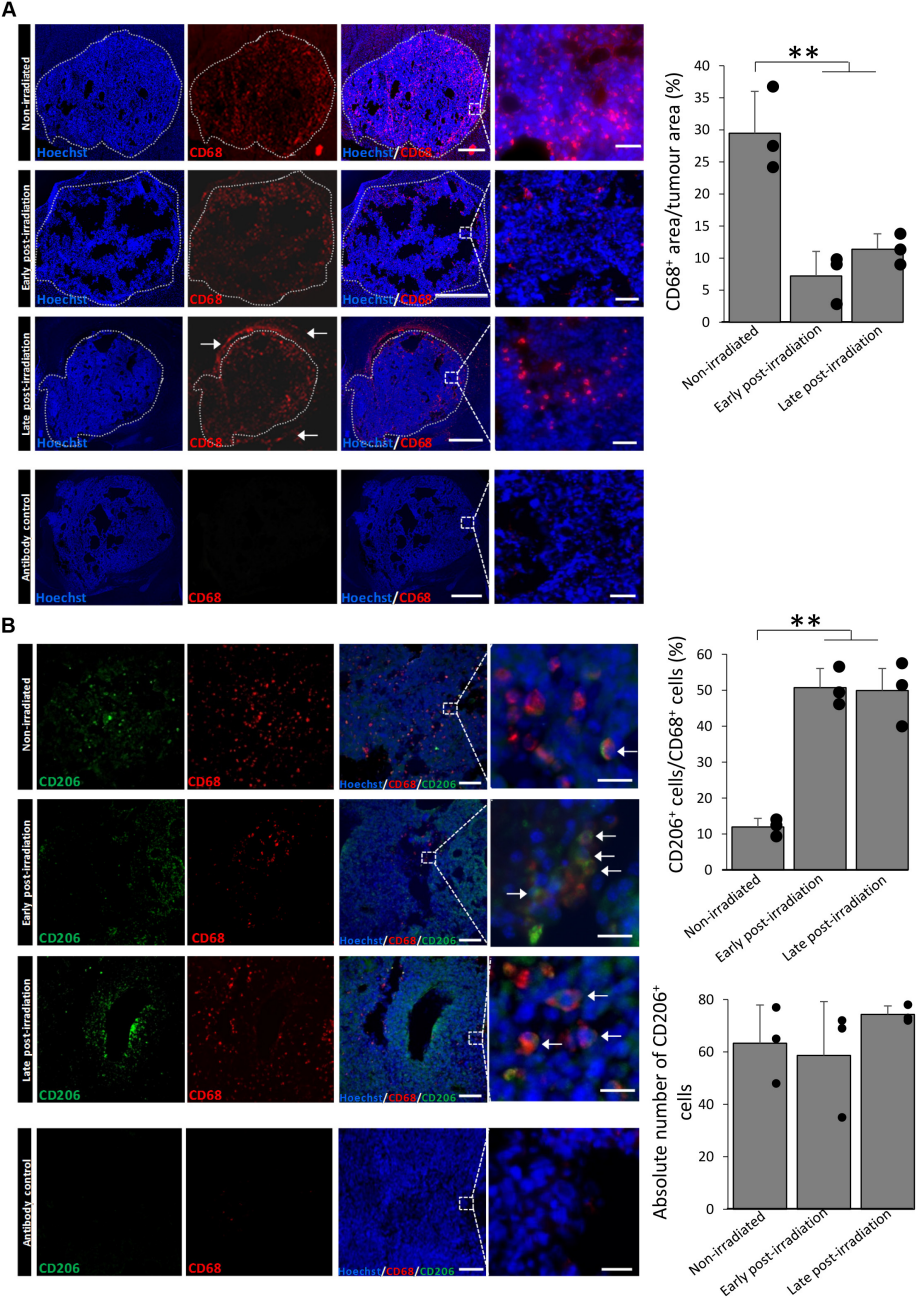
X-ray radiation increases the proportion of M2 MΦ in the GL261 GB model **(A)** Representative CD68 (red) and Hoechst 33342 (blue) immunofluorescent images of the GL261 orthotopic tumor model and the quantification of the percentage of CD68^+^ pixels compared to the tumor area before and early (3 days) or late (16 days) after X-Ray treatment. Scale bars=1000 μm or 50 μm for low or high magnification, respectively. Doted lines delimit the tumor areas from the brain tissue and they correspond to the areas of quantification. Black areas correspond to necrotic tissues and they are excluded from the quantification. White arrows indicate the CD68^+^ cells outside the tumor and which were not quantified. Antibody control images were used to confirm the specificity of the CD68 signal. **(B)** Representative CD206 (green), CD68 (red) and Hoechst 33342 (blue) immunofluorescent images of the GL261 orthotopic tumor and the quantification of CD206^+^ cells compared to CD68^+^ cells before and early (3 days) or late (16 days) after X-ray treatment. Scale bars=100μm for low magnification and scale bars=20μm for high magnification. Antibody control images were used to confirm the specificity of the CD206 and CD68 signals. Three sections for each animal and 3 animals per group were used (n=9 images per group, each point represent the mean of the 3 images), statistical significance was achieved when p<0.01(**).

We first aimed to determine whether X-ray radiation could influence MΦ present in GB. For that, GL261 GB-bearing mice were exposed to X-rays 7 days after cells implantation. Early post-irradiation treated animals and their respective controls were euthanized 14 days after cells implantation (3 days after the last IR) and late post-irradiation animals were euthanized 27 days after cells implantation (16 days after the last IR) (Figure 1). MΦ were detected by CD68 immunostaining. CD68^+^ cells were observed in the tumor mass of both non-irradiated and irradiated mice (Figure 2A). In non-irradiated mice, the density of CD68^+^ cells present in the core of the tumor was about 29.5±6.5% of the tumor area. However, after X-ray radiation, a significant decrease in CD68^+^ cells was observed. The density of CD68^+^ cells was 7.2±3.8% and 11.4±2.4% in the tumor mass early and late after X-ray treatment, respectively. However, in the late post-irradiation tumors, we can detect the presence of CD68^+^ cells outside the tumor core (white arrows). This suggests that CD68^+^ cells start to be recruited within the tumor. We then evaluated the percentage of M2 MΦ before and after IR by CD206 immunostaining (Figure 2B). While CD206^+^ cells represented about 12.0 ± 2.4 % of the CD68^+^ cells in the non-irradiated group, it significantly increased to 50.7 ± 5.3% and 49.9 ± 6.1 % in the tumor mass early and late after radiation, respectively, without any change in the absolute number of M2 MΦ (Figure 2B).

These results indicate that X-ray radiation decreases the number of MΦ but favors an enrichment in M2 phenotype in GB. We then asked the question, using *in vitro* experiments, whether the present observations were the result of a change in the MΦ phenotype after X-ray treatment or whether the difference was the results of preferential radio-sensitivity between MΦ phenotypes.

### X-ray radiation does not change the phenotype of MΦ

**Figure 3:**
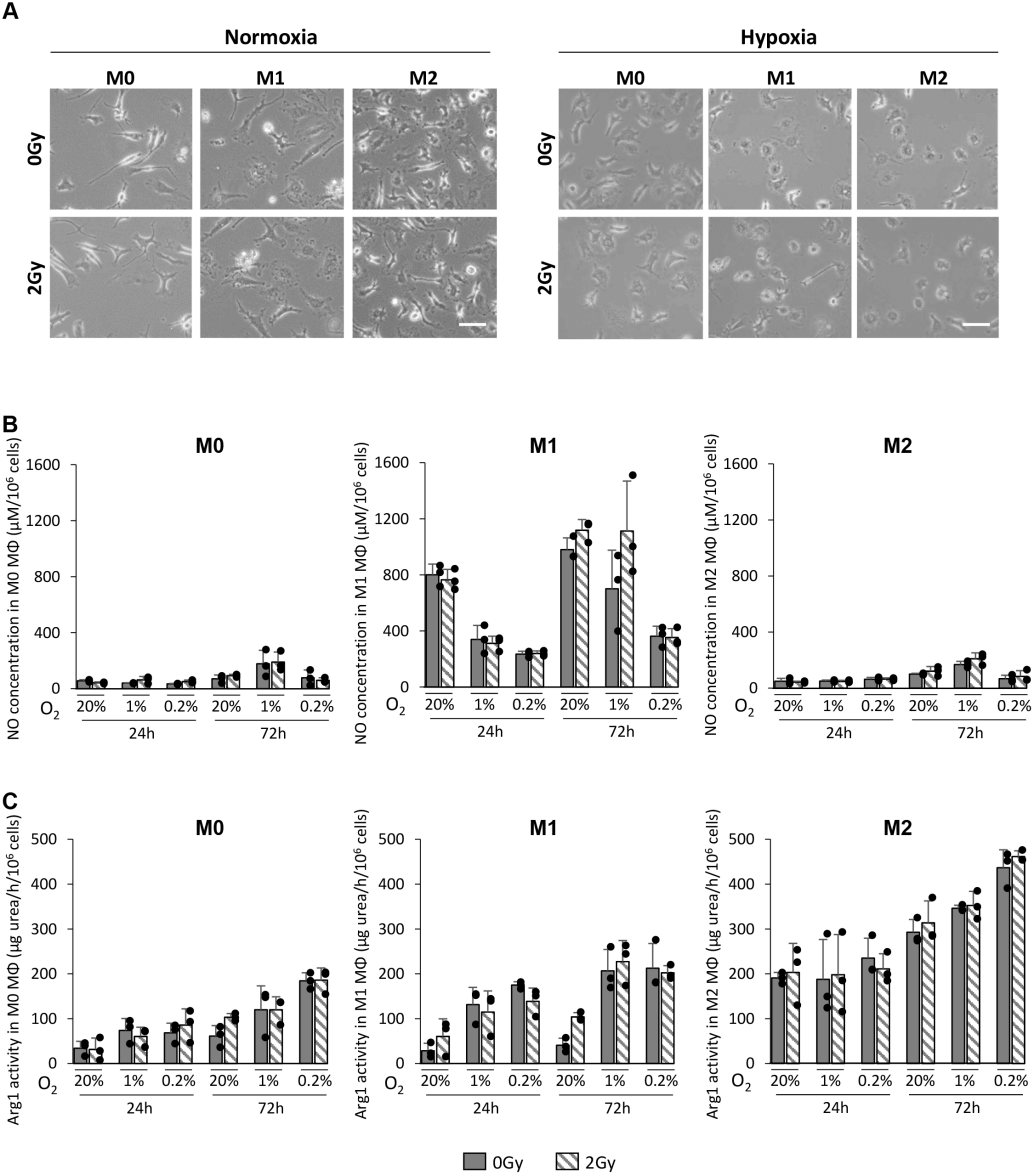
X-ray radiation does not change the phenotype of MΦ *in vitro* **(A)** Representative phase contrast microscopy images of M0, M1 and M2 MΦ 24h after 0Gy and 2Gy radiation in 20% (normoxia) or 0.2% O_2_ (hypoxia). Scale bar=20μm. **(B)** NO concentration (μM per 10^6^ cells) in M0, M1 and M2 MΦ 24h and 72h after 0Gy and 2Gy in 20%, 1% and 0.2% O_2_. **(C)** Arg1 activity (μg urea/h per 10^6^ cells) in M0, M1 and M2 MΦ 24h and 72h after 0Gy and 2Gy radiation in 20%, 1% and 0.2% O_2_. n=3 different experiments per condition.

We then aimed to determine whether X-ray radiation could change the MΦ phenotype in normoxic or hypoxic conditions. *In vitro*, M0, M1 and M2 MΦ were cultured in 20%, 1% or 0.2% O_2_ and cells were irradiated with a dose of 2Gy. From microscopic observation, no change at the morphological level was observed in any of the MΦ phenotypes after exposure to X-ray radiation in normoxia and in hypoxia (Figure 3A). To confirm this observation, the NO production (used as a marker of M1 MΦ) and the Arg1 activity (used as a marker of M2 MΦ) were analyzed 24h and 72h post-radiation, as we previously provided evidence that theses markers are robust markers to assess the phenotype of bone marrow derived MΦ [12]. As we published [12], NO was difficult to detect in M0 and M2 MΦ both in normoxia and hypoxia. In contrast, NO was easily detectable in M1 MΦ and the production decreased in hypoxia (Figure 3B). For all three phenotypes, X-ray radiation did not change NO production (Figure 3B). In parallel, Arg1 activity was weak in M0 and M1 MΦ in normoxia but was increased in both phenotypes when cultured under hypoxic conditions. There was a marked production of Arg1 in M2 MΦ in normoxia and, as expected, its activity was reinforced in hypoxia (Figure 3C) [12]. However, for the three phenotypes, X-ray radiation did not change the level of Arg1 activity either in normoxia or in hypoxia (Figure 3C). These results indicate that, in these experimental conditions, IR does not provoke direct changes in MΦ phenotypes under both normoxic and hypoxic conditions. Similar results were obtained with a high dose irradiation (8Gy) (data not shown).

### M0 and M1 MΦ are more sensitive to X-ray radiation than M2 MΦ

**Figure 4:**
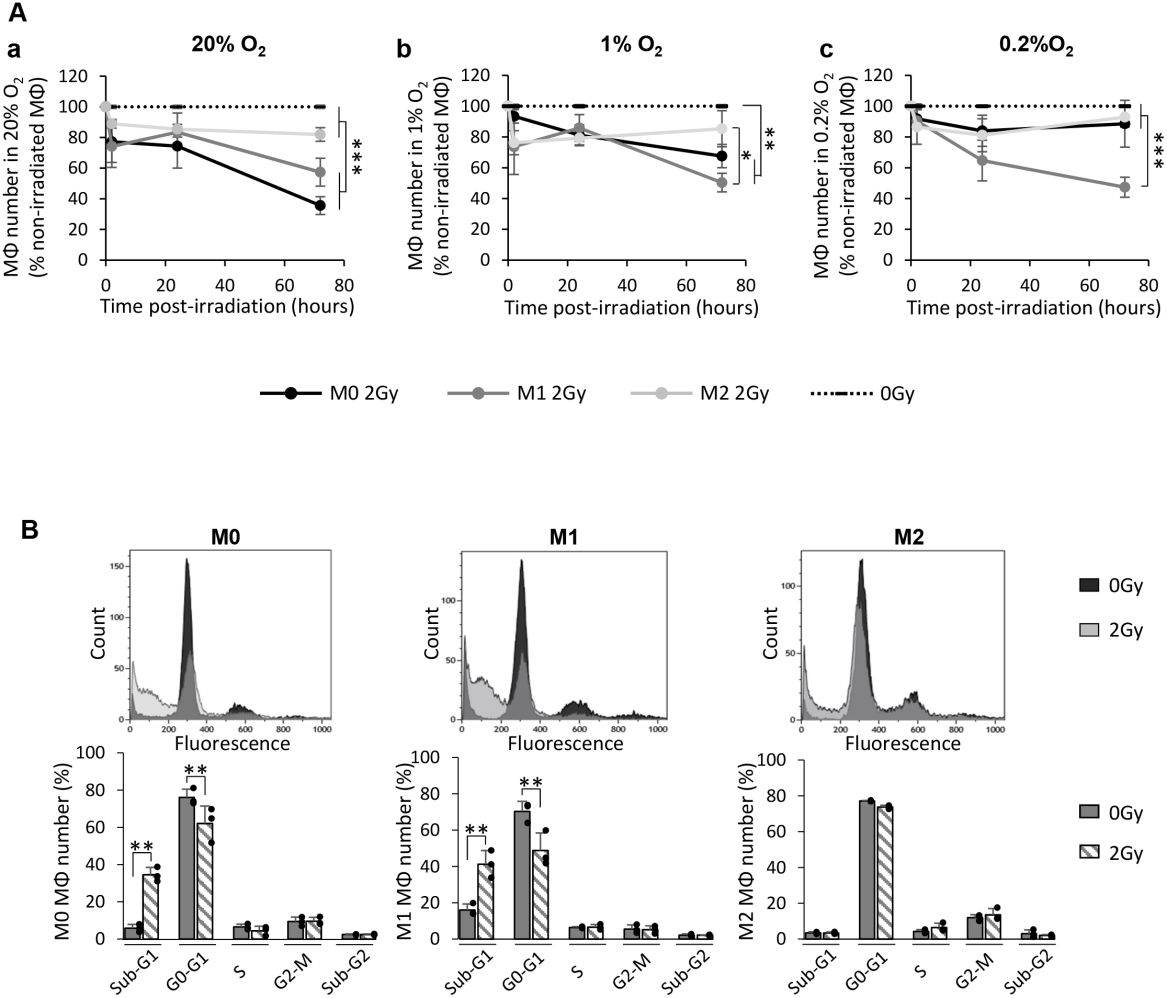
X-ray radiation induces cell death of M0 and M1 MΦ but not that of M2 MΦ *in vitro* **(A)** Kinetics (2h, 24h and 72h post-radiation) of M0, M1 and M2 MΦ cell numbers expressed as a percentage of control (0Gy) after 2Gy in 20% (a), 1% (b) or 0.2% O_2_ (c). Dotted lines correspond to non-irradiated M0, M1 and M2 MΦ. n=3 distinct experiments for each time point and each MΦ phenotype. Tukey's HSD test after significant one factor ANOVA (group) was used. Statistical significance was achieved when p<0.05(*), p<0.01(**) and p<0.001(***). **(B)** Cell cycle profiles and quantification of the cell distribution in different phases for M0, M1 and M2 MΦ 72h after 0Gy and 2Gy in 20% O_2_. n=3 different experiments per condition. Statistical significance was p<0.01(**).

The radiosensitivity of MΦ to X-rays was then evaluated by quantifying the cell number at different times post-radiation (2h, 24h and 72h) in 20%, 1% or 0.2% O_2_ (Figure 4A). In the 20% O_2_ condition (Figure 4Aa), the kinetic curves of M0 and M1 MΦ were significantly different from their respective control; an effect which was not observed for the M2 MΦ. The difference between kinetic curves of M0 and M1 MΦ numbers was not significant while both curves were significantly different from the M2 MΦ population decrease. At 72h post-radiation, only 35.6±5.8% of M0 MΦ and 57.3±9.1% of M1 MΦ were viable while about 81.9±4.4% of M2 MΦ were still detected (p<0.001 vs M0 and M1 MΦ). The changes in M0 and M1 MΦ survival, compared to M2 MΦ, were not due to a greater M2 MΦ proliferation (Supplementary Figure 1) but rather to a preferential cell death of M0 and M1 MΦ. Similar results were observed when the three phenotypes were irradiated with a dose of 8Gy (Supplementary Figure 2A). Furthermore, the decrease in M1 number following radiation was not influenced by hypoxia (1% and 0.2% O_2_) (Figure 4Ab and 4Ac). Indeed, the number of irradiated M1 MΦ were significantly reduced in all conditions, whatever the level of oxygenation, compared to non-irradiated M1 MΦ (at 72h post-radiation, only 50.3±6.1% and 47.3±6.5% of M1 MΦ were still viable in 1% and 0.2% O_2_, respectively). For M2 MΦ, all hypoxic conditions remained non-significant relative to non-irradiated conditions (85.4±11.7% and 92.9±6.5% of M2 MΦ were viable in 1% and 0.2% O_2_, respectively, 72h post-irradiation) (Figure 4Ab and 4Ac). However, M0 MΦ appeared less sensitive to IR when they were cultured in severe hypoxia (0.2% O_2_) since non-significant changes in cell survival were observed relative to M0 non-irradiated (at 72h post-irradiation, 88.5±15.2% of M0 MΦ were viable in 0.2% O_2_ compared to 67.6±7.6% in 1% O_2_) (Figure 4Ac). Hence, a hypoxia-dependent radioresistance was only highlighted for M0 MΦ while M2 MΦ remained resistant whatever oxygen concentration.

Cell death in normoxia in M0 and M1 MΦ was then confirmed by cell cycle studies performed by flow cytometry (Figure 4B). Only about 10% of non-irradiated M0 and M1 MΦ were found in the sub-G1 phase of the cell cycle, the cycle phase corresponding to cells with a DNA quantity inferior to 2n, which could be a reflection of cell death. However, a significant proportion of M0 (34.5±3.9%) and M1 (41.2±7.5%) MΦ was found in the sub-G1 phase of the cell cycle 72h after radiation. It could be noted that these changes in sub-G1 phase after irradiation is to the detriment of M0 and M1 MΦ proportion in G0/G1 phase. Concerning M2 MΦ, the cytometry profile confirmed the absence of an effect of IR on the cell cycle. Similar results were obtained with a high dose irradiation (8Gy) (Supplementary Figure 2B). M0 and M1 MΦ cell death were also confirmed by the increase in cell debris after IR (Supplementary Figure 3). These results support the hypothesis that M2 MΦ are more resistant to X-ray radiation compared to the M0 and M1 MΦ phenotypes. Given that 2Gy and 8Gy irradiations have similar effects on MΦ survival, only the results with the dose of 2Gy are presented thereafter.

### X-ray radiation induces similar DNA double-strand breaks between the different MΦ phenotype

**Figure 5:**
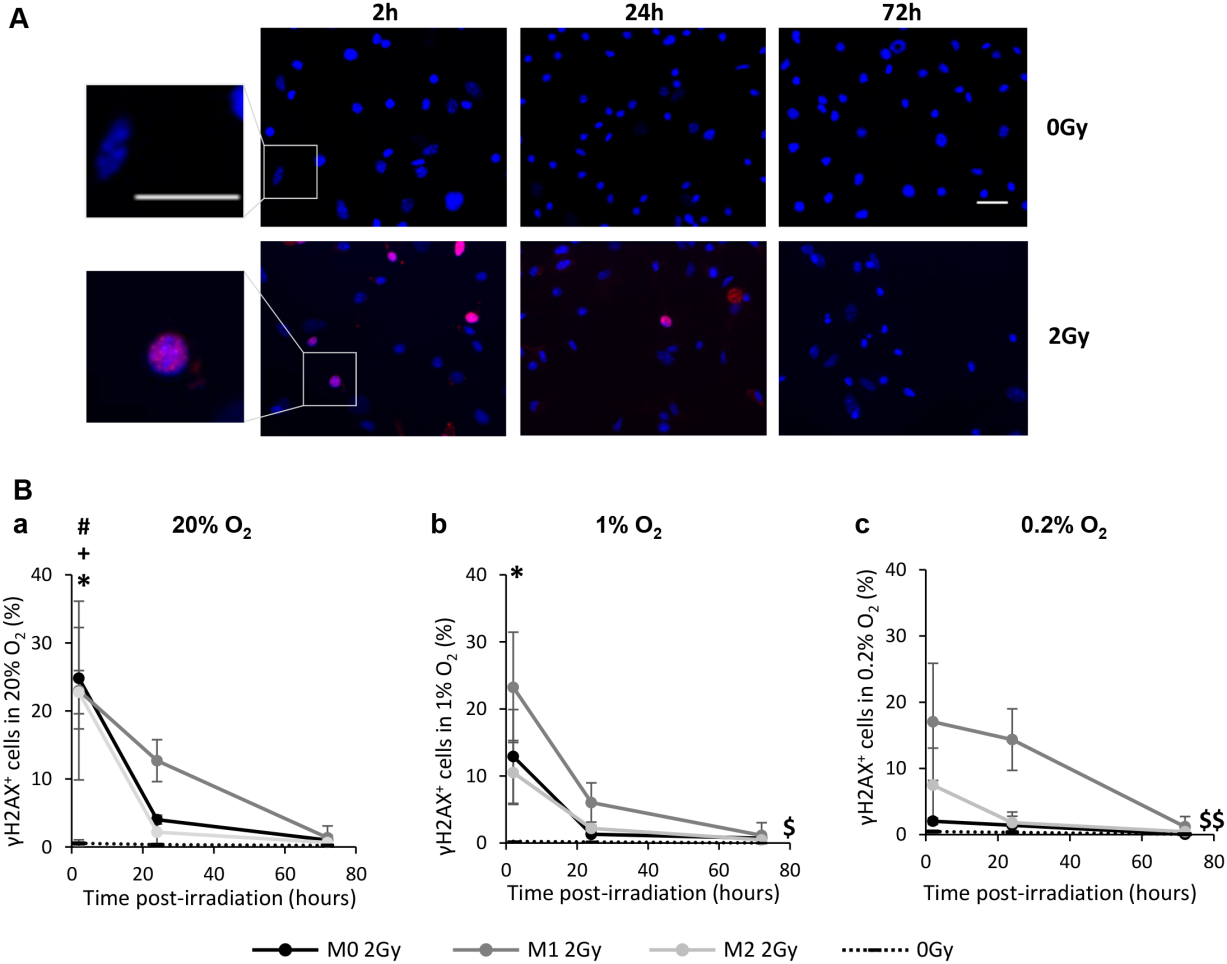
X-ray radiation generates DNA double strand breaks (DSBs) in the three MΦ phenotypes *in vitro* **(A)** Representative γH2AX and Hoechst 33342 immunofluorescence images of M0 MΦ 2h, 24h and 72h after 0Gy and 2Gy radiation in 20% O_2_. Scale bars=20μm. **(B)** Kinetics (2h, 24h and 72h post-radiation) of DNA DSBs (γH2AX^+^ cells) on M0, M1 and M2 MΦ after 2Gy in 20% (a), 1% (b) and 0.2% O_2_ (c). Dotted lines correspond to the mean of γH2AX^+^ cells in non-irradiated M0, M1 and M2 MΦ. n=3 experiments for each time point and each MΦ phenotype. Tukey's HSD test after significant one factor (group) or two factors ANOVA (time and group) were used. p<0.05(*) M0 2Gy vs M0 0Gy, p<0.05(#) M1 2Gy vs M1 0Gy and p<0.05(+) M2 2Gy vs M2 0Gy only 2h post-radiation. p<0.05($) and p<0.01($$) M1 kinetic curve vs M1 0Gy, M0 2Gy and M2 2Gy kinetics curves.

To decipher the mechanisms involved in the selective death of M0 and M1 MΦ, we analyzed the formation of DNA double strand breaks (DSBs) after irradiation. We evaluated whether M0, M1 and M2 MΦ respond to X-ray radiation in a similar way, both in normoxia and hypoxia. To evaluate DNA DSBs in the three MΦ phenotypes, γH2AX immunostaining was performed at different time post-radiation (2h, 24h and 72h) (Figure 5A) and the percentage of γH2AX^+^ cells (i.e. cells with more than 10 foci) was quantified (Figure 5B). Under normoxia, for the three MΦ phenotypes, the percentage of γH2AX^+^ cells was maximal 2h after 2Gy X-ray radiation (24.8±7.4% in M0 MΦ, 22.9±13.1% in M1 MΦ and 22.8±3.17% in M2 MΦ) and significantly different from the respective controls (0Gy, 0.6±0.5% of cells with DNA DSBs) (Figure 5Ba). While M1 MΦ present more γH2AX^+^ cells than M0 and M2 MΦ 24h after IR, this difference is not significant (p=0.5717 between M1 and M0 MΦ; p=0.3369 between M1 and M2 MΦ). Moreover, for the three phenotypes, the number of γH2AX^+^ cells decreases as a function of time to reach the level of the control cells 72h post-radiation. These results suggest that the genotoxicity induced by X-rays is similar between the three MΦ phenotypes and that they can repair DNA DSBs in normoxia.

In presence of 1% O_2_(Figure 5Bb), M0 MΦ present less γH2AX^+^ cells (12.9±6.9%) than in the 20% O_2_ condition but the difference is still significantly different from the control. M2 MΦ did not present significant number of γH2AX^+^ cells (10.5±4.7%) compared to control. However, the formation of DNA DSBs in M1 MΦ was not influenced by moderate hypoxia (23.2±8.2% of cells) and the kinetic curve of M1 MΦ was significantly different from the ones of M0 and M2 MΦ. This phenomenon was more pronounced at 0.2% O_2_ (Figure 5Bc). At 0.2% O_2_, the formation of DSBs was almost absent in M0 MΦ and significantly different from the 20% O_2_ condition (p=0.0317). These results suggest that in hypoxia, M1 MΦ are more sensitive to IR than M0 and M2 MΦ which present an important hypoxia-induced radioresistance.

To further elucidate the more pronounced radiosensitivity of M0 and M1 MΦ than M2 MΦ (Figure 4), we then focused on radio-induced cell death by studying apoptosis and mitotic catastrophe.

### M0 and M1 MΦ do not undergo apoptosis but manifest mitotic catastrophe after X-ray radiation

**Figure 6:**
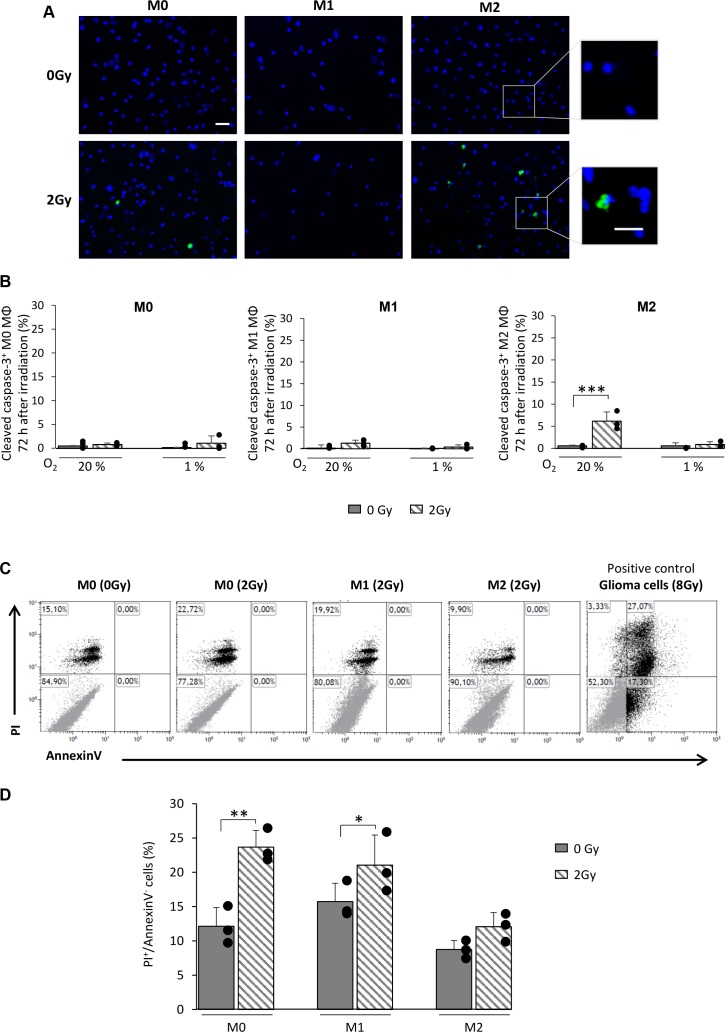
X-ray radiation fails to give rise to apoptosis in M0 and M1 MΦ *in vitro* **(A)** Representative cleaved-caspase-3 and Hoechst 33342 immunofluorescence images of M0, M1 and M2 MΦ 72h after 0Gy and 2Gy radiation in 20% O_2_. Scale bar=20μm for low magnification and scale bar=10μm for high magnification. **(B)** Quantification of cleaved-caspase-3 on M0, M1 and M2 MΦ 72h post-radiation (0Gy and 2Gy) in 20% and 1% O_2_. n=3 experiments per condition. Statistical significance was seen with the irradiated M2 MΦ [p<0.001(***)], otherwise no significant changes were noted. **(C)** Representative propidium iodide (PI)/AnnexinV flow cytometry profiles of non-irradiated M0 MΦ (0Gy) and M0, M1 and M2 MΦ 72h after irradiation (2Gy). Irradiated glioma cells (8Gy) were used as positive control for AnnexinV staining. **(D)** Quantification of IP^+^/AnnexinV^−^ cells in M0, M1 and M2 MΦ 72h after 0Gy and 2Gy radiation. n=3 different experiments per condition. Statistical significance was achieved when p<0.05(*) and p<0.01(**).

According to the cell cycle profiles of M0 and M1 MΦ after X-rays (Figure 4B), we first posed the question whether the increase in sub-G1 phase is an index of apoptosis. We analyzed the activation of the cleaved form of caspase-3 in the three MΦ phenotypes 72h post-radiation (Figure 6A). M0 and M1 MΦ failed to show a difference in the number of cleaved caspase-3^+^ cells after irradiation compared to control cells. However, a small population of M2 MΦ (7.2±2.3%) showed a cleaved caspase-3^+^ after radiation but only when the cells were cultured in 20% O_2_ (Figure 6B). To further reinforce this result, Propidium Iodide (PI)/AnnexinV flow cytometry was performed on the three MΦ phenotypes (Figure 6C). No AnnexinV^+^ cells were detected following irradiation whatever the phenotype studied, supporting the absence of apoptotic death after IR. Increased PI staining in M0 and M1 MΦ is indeed in favor of a radio-induced mitotic catastrophe (Figure 6C and 6D).

**Figure 7:**
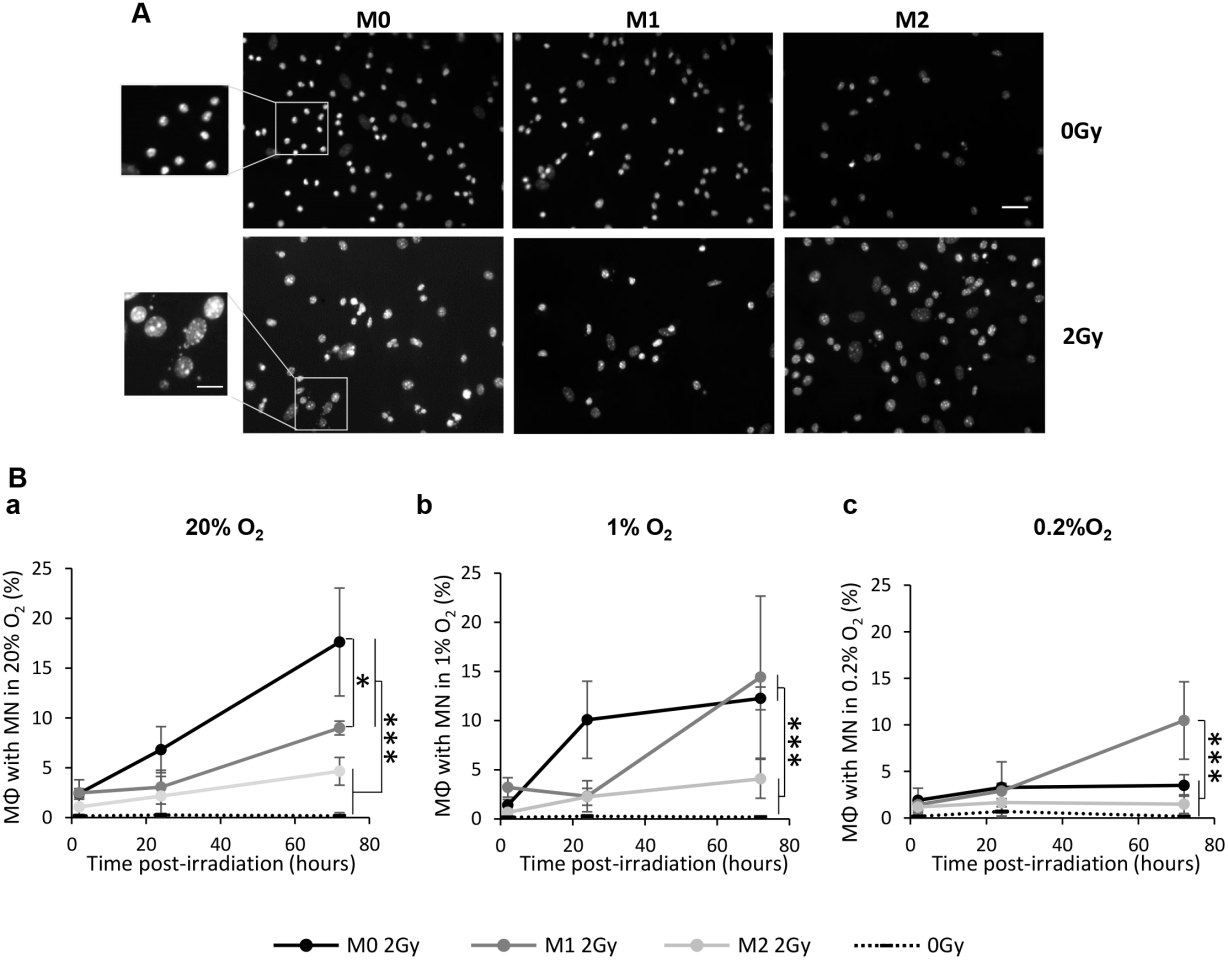
X-ray radiation induces the mitotic catastrophe in M0 and M1 MΦ *in vitro* **(A)** Representative Hoechst 33342 immunofluorescence images of M0, M1 and M2 MΦ 72h after 0Gy and 2Gy radiation in 20% O_2_. Scale bar=20μm for low magnification and scale bar=10μm for high magnification. **(B)** Kinetics (2h, 24h and 72h post-radiation) of percentage of M0, M1 and M2 MΦ with micronuclei (MN) after 2Gy radiation in 20% (a), 1% (b) and 0.2% O_2_ (c). Dotted lines correspond to the mean of cells with MN in non-irradiated M0, M1 and M2 MΦ. n=3 experiments for each time point and each condition. Tukey's HSD test after significant one factor ANOVA (group) was used. Statistical significance were p<0.05(*) and p<0.001(***), otherwise it was not significant.

To confirm the radio-induced mitotic catastrophe in M0 and M1 MΦ, we analyzed the formation of micronuclei (MN), as a reflection of aneuploidy following genomic instabilities [23] (Figure 7A). M0, M1 and M2 MΦ were irradiated in 20%, 1% and 0.2% O_2_ and the MN were counted at different times post-radiation (2h, 24h and 72h) (Figure 7B). At 72h post-radiation, IR resulted in a significant increase in the percentage of MN positive cells (i.e. cells with at least one MN) for both M0 and M1 MΦ in 20% (17.6±5.4% for M0 MΦ and 8.9±0.7% for M1 MΦ with MN) (Figure 7Ba) and 1% O_2_ (12.3±1.1% for M0 MΦ 14.4±8.2% for M1 MΦ) (Figure 7Bb) relative to non-irradiated cells. M2 MΦ presented only 4.6±1.4% of cells with MN at 20% O_2_ and 4.0±1.9% at 1% O_2_, differences which were not significantly different from control cells (Figure 7Ba and 7Bb). Interestingly and in line with previous results concerning the cell number, in M0 MΦ, the radiation-induced MN formation was significantly less when the cells were cultured at 0.2% O_2_ compared to 20% and 1% O_2_(Figure 7Bc). However, M1 MΦ still present MN formation 72h post-irradiation at 0.2% O_2_ (10.5±4.1% of cells with MN) which is significantly different to respective control, and irradiated M0 and M2 MΦ (Figure 7Bc).

Altogether, these results indicate that M0 and M1 MΦ do not undergo apoptosis following IR but rather radio-induced mitotic catastrophe. Moreover, it seems that M1 MΦ is the most radiosensitive phenotype since these cells react similarly in both normoxia and in hypoxia.

## DISCUSSION

MΦ, the most abundant inflammatory cells found in GB [5], promote tumor development and represent a negative prognostic factor [24] notably because of the presence of the M2 phenotype [8]. However, the impact of conventional therapies, and particularly radiotherapy, on these cells is still not well understood. We have shown, *in vivo*, that radiotherapy with X-ray exposure induces a loss of MΦ present in GB. Interestingly, we have demonstrated that the proportion of M2 MΦ (CD206^+^ cells) relative to total MΦ (CD68^+^ cells) was increased after IR. *In vitro*, we have confirmed that these changes are not due to a modification of the MΦ phenotype but rather to an increase in the M2 MΦ population by preferentially inducing cell death of M0 and M1 MΦ. In contrast to M0 and M1 MΦ, M2 MΦ are less sensitive to irradiation.

We describe a decrease in MΦ number occurring early after IR as already mentioned in prostate cancer [17]. In the late time, the presence of CD68^+^ cells outside the tumor core was also observed suggesting that CD68^+^ cells start to be recruited within the GB tumor in good agreement with the literature in GB [13, 27] and other tumors [16].

Concerning the MΦ phenotype, several controversial investigations have analyzed the impact of IR on MΦ phenotype *in vivo*. For instance, an increase in M1 MΦ markers was noted after radiation therapy [25–27] while others have reported an increase in M2 MΦ markers [15, 28, 29]. Moreover, a lack of effect of radiation on cytokine production was observed *in vitro* [19, 30]. In contradistinction, we have shown that MΦ in GB change their morphology and an increase in M2 marker is observed after IR, as previously described [17]. However, in this study, we have found that X-ray treatment increased the M2 MΦ proportion in a GB model of recurrence. Two main hypotheses have been proposed to elucidate how IR influences MΦ: i) X-ray exposure alters the phenotype of MΦ; or ii) one phenotype is more resistant to radio-induced cell death than the others.

Within the context of these two hypotheses, we have demonstrated *in vitro* that the phenotype of MΦ remained unchanged with X-ray radiation. NO and Arg1 were used as we previously provided evidence that theses markers are sensitive markers of bone marrow derived MΦ [12]. These results led us to postulate that the sensitivity to X-ray radiation could be different depending on the MΦ phenotype.

Controversial and contradictory studies related to the impact of radiation therapy on MΦ fate have been published. While numerous studies have described MΦ as a radioresistant cell type [19, 30], other studies have found either an increase in MΦ in the tumor following X-ray or a decrease [15, 18]. However, no single investigation has differentiated the response of the three distinct phenotypes to direct radiation. In the present study, we have found that M0 and M1 MΦ were more sensitive to radiation than M2 MΦ.

An important decrease in the number of M0 and M1 MΦ was observed *in vitro* in 20% O_2_, as these phenotypes are usually found in oxygenated areas of GB [12], following radiation. This observation was also noticed when the cells were cultured under moderate hypoxic condition (1% O_2_). Although we have shown that M0 and M1 MΦ are able to repair DNA DSBs, their number also decreased post-radiation. This profile might suggest that M0 and M1 MΦ misrepair DNA DSBs leading to certain genomic instabilities. Both nuclear fragmentation (sub-G1 phase) and MN formation along with the absence of cleaved-caspase 3^+^ cells and AnnexinV^+^ cells favor to conclude for the presence of radio-induced mitotic catastrophe in these two phenotypes after IR. However, in severe hypoxia (0.2% O_2_), M0 MΦ number remained stable after IR while M1 MΦ were still decreased. One possible explanation at the decrease in DNA DSBs for M0 MΦ at 0.2% O_2_ could be that at these low O_2_ pressure, M0 MΦ are already engaged toward an M2 phenotype, as already described [12]. For M1 MΦ, the DNA DSBs and the MN profiles confirm that these cells responded to IR in severe hypoxia. Considering the effect of X-rays on M0 MΦ 2h after treatment and the fact that irradiation is affected by hypoxia, these results would suggest a role of reactive oxygen species (ROS) in the process of radio-induced M0 MΦ death. Indeed, it was described that IR induced an important production of ROS, almost instantly after treatment, which could contribute to genomic instability [31]. Moreover, hypoxia is known to reduce ROS accumulation [32] which correlates with the decrease in M0 MΦ death in oxygen-deprived environment. However, this last phenomenon was not observed in M1 MΦ, suggesting another mechanism involved and more particularly the NO, as M1 MΦ are known to produce large amount of NO [33]. Indeed, NO can induce radiosensitization of cells under hypoxic conditions [34–36], including GB cells [37, 38] by enhancing DNA DSBs [38], limiting DNA repair [39] and inducing mitotic catastrophe [37]. This could explain the mechanism involved in M1 radio-induced cell death as we have shown that M1 MΦ produced important quantity of NO which decreased in hypoxia, as already published [12], but still superior to the amount observed in M0 and M2 MΦ. This effect could correlate with the important M1 MΦ death observed in normoxia, which was reduced in hypoxia but still present.

Concerning M2 MΦ, their number was not impacted by radiation and MN formation was not found 72h post-radiation. While M2 MΦ exhibited DNA DSBs, these data allow us to suggest that M2 MΦ are able to faithfully repair DNA DSBs and are more radioresistant to X-rays. The proportion of apoptotic cells in the M2 phenotype was low (7%) and no AnnexinV^+^ cells were observed. All together, these results suggest that M2 MΦ are radioresistant. Interestingly, cell death failed to occur on M2 MΦ in hypoxic conditions, a situation in which M2 MΦ are formed to the detriment of M0 and M1 MΦ [12]. To understand why M2 MΦ are more radioresistant, we performed western blot analyses on P-Erk/pan-Erk and P-Akt/pan-Akt known to be two major players in radioresistance and observed increased expression of P-Erk and P-Akt only in M2 relative to M0 and M1 macrophages (not shown). However, at the present time, we cannot rule out that many other intracellular players could explain this resistance.

Altogether, our *in vitro* study supports the hypothesis that radiation therapy could increase the proportion of M2 MΦ in the tumor. These data concur with the observation of Chiang and colleagues who reported, on animals bearing brain tumors, an increased proportion of the M2 phenotype [17]. Based on our observations, we have made the hypothesis that such a selection may also occur due to the M0 and M1 MΦ death, in parallel to a synthesis of polarizing cytokines.

Despite various and vigorous treatments, recurrence always occurs in GB [40]. The presence of glioblastoma stem cells (GSC) has been proposed as a potential explanation for this recurrence [41]. Following our observations, one might propose that the enrichment of the M2 phenotype after radiotherapy could also promote GB recurrence. In accordance with this hypothesis, M2 MΦ are known to associate themselves with GSC in hypoxic areas [42, 43] to promote tumor development [44]. Moreover, it has been described that the recruitment of myeloid cells following radiation is also responsible for recurrence in different tumors [45, 46]. In the immediate phase after IR, the number of MΦ decreased before the recruitment, approximately 20 days post-radiation [13]. This MΦ repopulation has been, in part, explained by the fact that IR can induce endothelial cell death [47, 48] which leads to the development of hypoxia [13, 49] and subsequently an increase in chemokines, such as SDF-1 [13, 14, 50]. The difference between our study and that of Chiang and collaborators [17] and Kioi and collaborators [13] is that they observed an increase in MΦ migration in parallel to an increase in M2 MΦ number after X-rays because their observations were made about 22 days after treatment, when MΦ are recruited in GB [13]. In our study, we observed an increase in the proportion of M2 MΦ (the phenotype relatively resistant to IR) after X-ray treatment just before MΦ recruitment.

The limits of this study are the lack of sensitive markers to differentiate M0, M1 and M2 MΦ on mouse brain slides but also the impossibility to perform clonogenic assays (standard colony formation or soft-agar colony formation assays). Another limitation of this study is the use of a single tumor model developed in the mice. It is however important to mention that this is a syngeneic immunoproficient model that also recapitulates various features of the human situation (hypoxia, invasion) [51–53]. Despite these caveats, the strength of this study relies on the first demonstration that the three MΦ phenotypes respond differentially to IR. This has led us to argue that the increase in M2 MΦ proportion in GB after X-ray treatment is not due to a switch in MΦ phenotypes but rather to the selective death of M0 and M1 MΦ. This pathophysiological process is important to take into account because M2 MΦ are known to promote tumor development and most recently, studies from the literature indicate that the different MΦ phenotypes can induce differential responses of tumor cells to various treatment options and, especially, different chemotherapies [21].

As a conclusion to our study, we have made the assumption that IR could differentially influence the three phenotype of MΦ found in tumors. We have demonstrated that the three phenotypes respond to radiation in a phenotype-specific manner. M0 and M1 MΦ phenotypes undergo a mitotic death following radiation thereby decreasing cell numbers while M2 MΦ were radioresistant especially in situations with low O_2_, areas in which they are mainly enriched [12, 54]. X-ray radiotherapy can contribute, along with other phenomena, to the increased density of M2 MΦ in GB.

## MATERIALS AND METHODS

### Cell cultures

The murine GB cell line, GL261[NCI-DCTD (Division of Cancer Treatment and Diagnosis) Repository], was cultured in Roswell Park Memorial Institute media (Sigma-Aldrich) supplemented with 10% fetal calf serum (Eurobio), 1μg/ml penicillin/streptomycin (P/S, Sigma-Aldrich) and 2mM of glutamine (Gln, Sigma-Aldrich) at 37°C in a humidified atmosphere and in mycoplasma free conditions.

Bone marrow-derived MΦ were obtained from mice [20-22g, CURB, Univ. Caen, France] and were isolated from femora and tibiae by flushing the bones with 1ml of Iscove's Modified Dulbecco's Media (IMDM, Sigma-Aldrich) containing 60% Fetal Clone II (FCII, Thermo Scientific) and 1μg/ml P/S. The marrow was passed through a 70μm strainer and MΦ (M0) were selected and cultured in IMDM enriched with 15% FCII, 1μg/ml P/S, 10 ng/ml recombinant mouse macrophage colony-stimulating factor (M-CSF, Miltenyi Biotec) and 10 ng/ml recombinant mouse Fms-related tyrosine kinase 3-ligand (Flt3-ligand, Miltenyi Biotec) at 37°C in a humid atmosphere. M1 MΦ were obtained by culturing cells in 1g/l glucose Dulbecco's Modified Eagle Medium (DMEM, Sigma-Aldrich) supplied with 15% FCII, 1μg/ml P/S, 2mM Gln (Sigma-Aldrich), 100 ng/ml LPS (Sigma-Aldrich) and 10U/ml recombinant mouse interferon-gamma (IFN-γ, eBioscience). M2 MΦ were obtained by culturing cells with 1g/l glucose DMEM supplemented with 15% FCII, 1% P/S, 2mM Gln (Sigma-Aldrich) and 50 ng/ml recombinant mouse interleukine 4 (IL4, Miltenyi Biotec).

### GB preclinical model

Tumor models consist of an orthotopic injection of GL261 cells in C57/Bl6 mice (20-22g, Janvier laboratories). The animal investigations were performed under the current European directive (2010/63/EU). The license to investigate was given to SV (14-55) in authorized housing and laboratories (B14118001) and with the permission of the regional committee on animal ethics (N/04-01-13/04/01-16). Mice were operated under anaesthesia (induction in 5% and maintenance in 2% of isoflurane in 70% NO_2_/30% O_2_) and GL261 cells were injected [1.10^5^ cells in 3μl in 2mM Gln/phosphate buffer saline (PBS)] in the right caudate-putamen.

### Hypoxic cell treatment

Normoxia (20% O_2_) cells were cultured in a humidified 5% CO_2_/air atmosphere. Moderate (1% O_2_, the O_2_ level commonly found in GB) [22] and severe (0.2% O_2_, the O_2_ level found around necrotic areas of GB) hypoxic cells were cultured in a humidified 5% CO_2_/balance N_2_ gas mixture in a hypoxic chamber (Invivo2 500, Ruskinn, Awel) at 37°C. For radiation treatment, M0 MΦ were cultured either in normoxia or in hypoxia 6h before radiation. M1 and M2 MΦ were activated for 24h and then cultured in normoxia or hypoxia 6h before radiation with their respective conditioning media. The hypoxic culture medium was equilibrated for 30 min with the gas mixture contained in the hypoxia chamber before add it to cell cultures.

### Radiation treatments

All radiation experiments were performed on the XRad225Cx (PXi, CYCERON platform).

For *in vivo* experiments, the radiation of ipsilateral hemisphere was realized seven days after the implantation of GB cells (tumor volume around 10mm^3^). Mice were anaesthetized as described above and irradiated thrice with 4Gy dose every two days at a dose rate of 3.3Gy/min. The animals were sacrificed 15 days after implantation of tumor cells for the non-irradiated animals and 7 days and 20 days after the first irradiation for the irradiated animals (Figure 1). The tumor volume was evaluated by a T2w MRI scan (7T MRI, Bruker, CYCERON Imaging platform).

For *in vitro* experiments, cells were exposed at room temperature to X-ray to a unique dose of 2Gy (or 8Gy) at a dose rate of 2Gy/min. After radiation, cells were maintained under normoxic or hypoxic conditions until the end of the experiment.

### Immunohistochemistry

At the end of the protocol, mice were deeply anaesthetized and were transcardially perfused with a 0.2M phosphate buffer (PB)/4% paraformaldehyde (PFA, Sigma-Aldrich). Brains were removed and placed in 30% sucrose for 48h and 30μm thick freezing microtome sections were realized. Slices were blocked with PBS, 0.5% Triton, 3% bovine serum albumin (BSA, Sigma-Aldrich) for 2h and then incubated overnight with anti-CD68 (1/800, Abcam, ab53444) and anti-CD206 (1/1000, Abcam, ab64693) antibodies in PBS, 0.5% Triton, 1% BSA at 4°C. Sections were then incubated with an Alexa-555-conjugated anti-rat (1/200, Invitrogen, A18744) or an Alexa-488-conjugated anti-rabbit (1/200, Invitrogen, 10424752) as secondary antibodies in PBS, 0.5% Triton, 1% BSA containing Hoechst 33342 (10μg/ml, Sigma-Aldrich).

### Immunocytochemistry

MΦ were fixed with a 0.2M PB/4% PFA solution. Cells were blocked with PBS, 0.1% Tween, 3% BSA for 30min and the cells were firstly incubated overnight at 4°C with the primary antibody in PBS 0.1% Tween, 1% BSA at 4°C. The following primary antibodies were used: phosphorylated histone H2AX (ser139) (γH2AX; 1/200; Cell Signalling Technology, 2577S) and cleaved-caspase-3 (1/600; Cell Signaling Technology, 9661S). γH2AX and cleaved-caspase-3 are sensitive markers of DNA double-strand breaks [55] and apoptosis, respectively. Cells then were incubated with an Alexa-555-conjugated anti-rabbit secondary antibody (1/200; Invitrogen, A31572) and Hoechst 33342 (10μg/ml) in PBS 0.1% Tween, 1% BSA for 2h at room temperature.

### Cytotoxic assay

The effect of radiation on cell survival was measured by manually counting the cell number 2h, 24h and 72h after radiation. MΦ were fixed as mentioned above and cell nuclei were stained with Hoechst 33342 (10μg/ml).

### Image analysis

Images were acquired thanks to the time-lapse microscope (DMi8 S imaging system, Leitz LEICA) and then analyzed by ImageJ software (http://imagej.nih.gov/ij/). For immunohistochemistry, MΦ density was determined as the number of positive area for CD68 divided by the total tumor area using an automatic thresholding. The percentage of M2 MΦ was determined by a manual counting of the number of cells that expressed both CD206 (M2 MΦ) and CD68 (MΦ). For immunocytochemistry, the number of cells was manually counted. For γH2AX immunostaining, cells with at least 10 foci in nucleus were considered as positive. The percentage of γH2AX^+^ cells was determined by the number of γH2AX compared to the total number of cells (Hoechst 33342). The percentage of cleaved-caspase 3^+^ cells was determined by the number of cleaved-caspase 3^+^ cells compared to the total number of cells (Hoechst 33342). The presence of micronuclei (MN, indicative of genomic instability followed by mitotic catastrophe [56]) was assessed by Hoechst 33342 staining and a cell with at least one MN was considered positive.

### Cell cycle analysis

The cell cycle of MΦ was studied by flow cytometry with the DNA-prep reagents kit according to manufacturer's instructions (Beckman Coulter SAS, France). Propidium iodide staining was analyzed by the Gallios^TM^ flow cytometer (Beckman Coulter SAS, France) with 20 000 events per determination. The analysis and determination of the cell distribution in each phase of the cell cycle was achieved based on the Kaluza® Flow Analysis software (Beckman Coulter SAS, France).

### Propidium iodide (PI)/AnnexinV analysis

This experiment was performed using Annexin V-FITC Kit (Beckman Coulter), following the manufacturer's protocol. Briefly, cell samples (M0, M1 and M2 macrophages irradiated or not) were washed with cold PBS and cell pellet was resuspended in 1X binding buffer and immediately kept on ice. Annexin V-FITC solution and Propidium Iodide (IP) were added and incubated for 15 minutes on ice in the dark. Subsequently, cells were analyzed by flow cytometry, using Gallios^TM^ flow cytometer and at least 20 000 events were collected per sample. Data was analyzed using Kaluza® Flow Analysis software.

### Determination of nitric oxide (NO) production

NO measurement in the supernatant of MΦ cultures was performed by the Griess reaction [57]. Each sample was assayed in duplicate, the absorbance was measured at 540 nm and the NO concentration was determined with sodium nitrite as a standard.

### Determination of Arg1 activity

Arg1 activity was determined by a standard colorimetric method in cell lysates as published [57]. Each sample was assayed in duplicate, the absorbance was measured at 540 nm and urea production was determined with urea as the standard.

### Online supplementary materials

Supplementary Materials about proliferation assay and cell debris analyses are available in the online version of the paper.

### Statistical analyses

Data are represented by the mean±standard deviation (SD), the circles representing results of individual experiment. Statistical analyses were performed with the JMP® program (SAS institute, USA) and, unless otherwise stated, significances were calculated by the Tukey's HSD test after significant ANOVAs.

## SUPPLEMENTARY MATERIALS FIGURES AND TABLES



## Abbreviations

Arg1: arginase 1
DSB: double strand breaks
GB: glioblastoma
GSC: glioblastoma stem cells
iNOS: inducible nitric oxide synthase
IR: ionizing radiation
MΦ: macrophages
MN: micronuclei
NO: nitric oxide
O2: oxygen
PI: propidium iodide
SDF-1: stromal cell derived factor 1
TAM: tumor associated macrophages
