# Supplementary material for: M2 macrophages are more resistant than M1 macrophages following radiation therapy in the context of glioblastoma
Source: Oncotarget. 2017 Aug 7;8(42):72597–612. doi: 10.18632/oncotarget.19994 (PMC5641155; doi:10.18632/oncotarget.19994)
Supplement: Supplementary file 1 [file oncotarget-08-72597-s001.pdf]

M2 macrophages are more resistant than M1 macrophages following radiation therapy in the context of glioblastoma

SUPPLEMENTARY MATERIALS

Proliferation assay

The proliferation rate was assessed by counting the cell numbers 1, 3 and 7 days after seeding. MΦ were fixed with a 0.2M PB/4% PFA solution and cell nuclei were stained with Hoechst 33342 (10 µg/ml). Cell numbers were counted by in-house macros based on ImageJ software. Each experiment was performed in triplicate.

Cell debris analysis

From the flow cytometry analyzes (Gallios™ flow cytometer, Beckman Coulter SAS, France) obtained with IP/AnnexinV staining, a biparametric histogram representing cell size (SS, side scatter) as a function of cellular granularity (FS, forward scatter) was obtained. Two distinct cell populations in terms of size could clearly be identified as dead cells (corresponding to cell debris) and living cells. The positioning of the gates as well as the quantification of the proportion of dead and living cells has been performed with the Kaluza® Flow Analysis software (Beckman Coulter SAS, France).

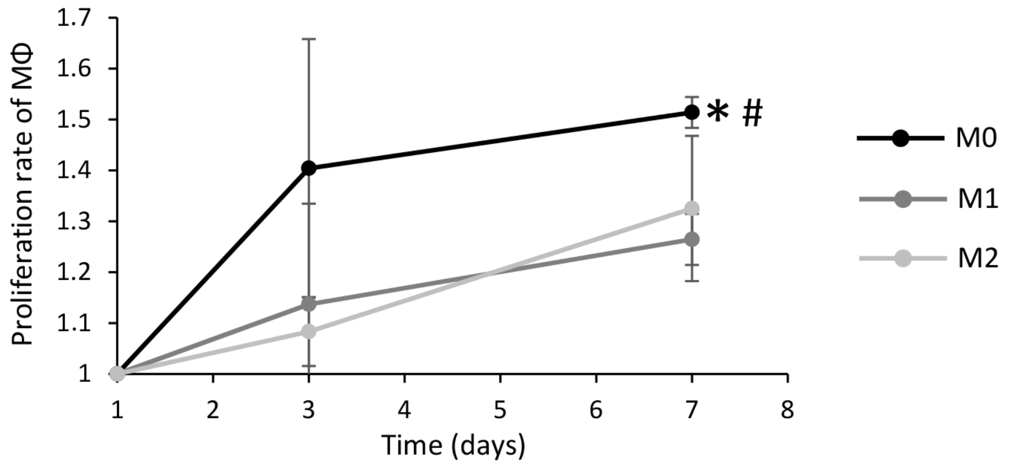

Supplementary Figure 1: Profile of MΦ proliferation. Kinetics (1, 3 and 7 days after seeding) of proliferation of M0, M1 and M2 MΦ. Mean ± SD, n = 3 for per time and per MΦ phenotype. $p<0.05$ (*) M0 vs M1, $p<0.05$ (#) M0 vs M2.

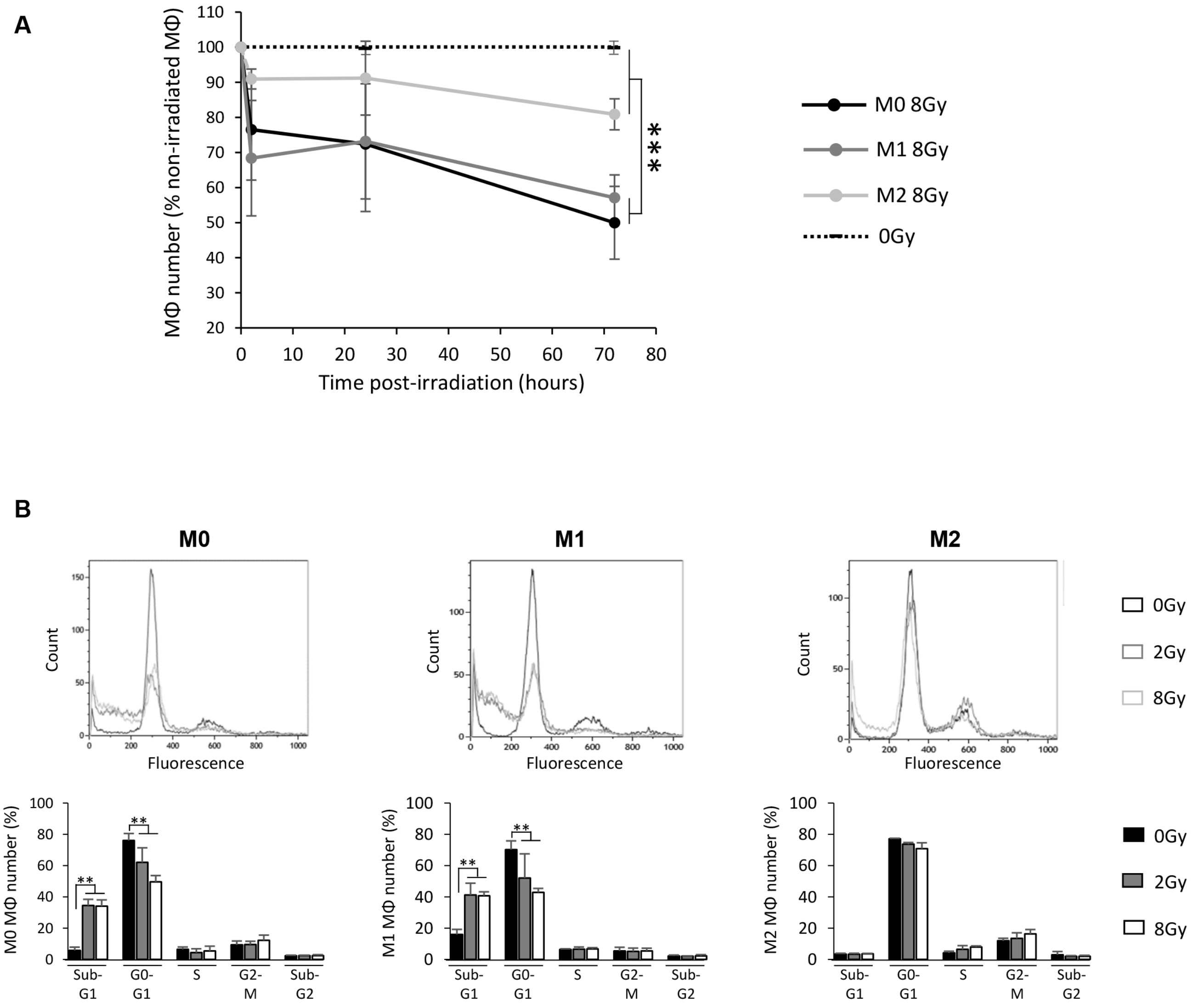

Supplementary Figure 2: MΦ cell death with 8Gy irradiation. (**A**) Kinetics (2h, 24h and 72h post-radiation) of M0, M1 and M2 MΦ cell numbers expressed as a percentage of control (0Gy) after 8Gy in 20% O_2. n=3 for each time point and each MΦ phenotype. Statistical significance was p<0.001 (***) M2 MΦ vs M0 and M1 MΦ, p<0.001 (###) M0 MΦ after 8Gy compared to its respective control and p<0.001 (+++) M1 MΦ after 8Gy compared to its respective control. (**B**) Cell cycle profiles and quantification of the cell distribution in different phases for M0, M1 and M2 MΦ 72h after 0Gy, 2Gy and 8Gy in 20% O_2. Mean ± SD, n=3 per condition. Statistical significance was p<0.01 (**).

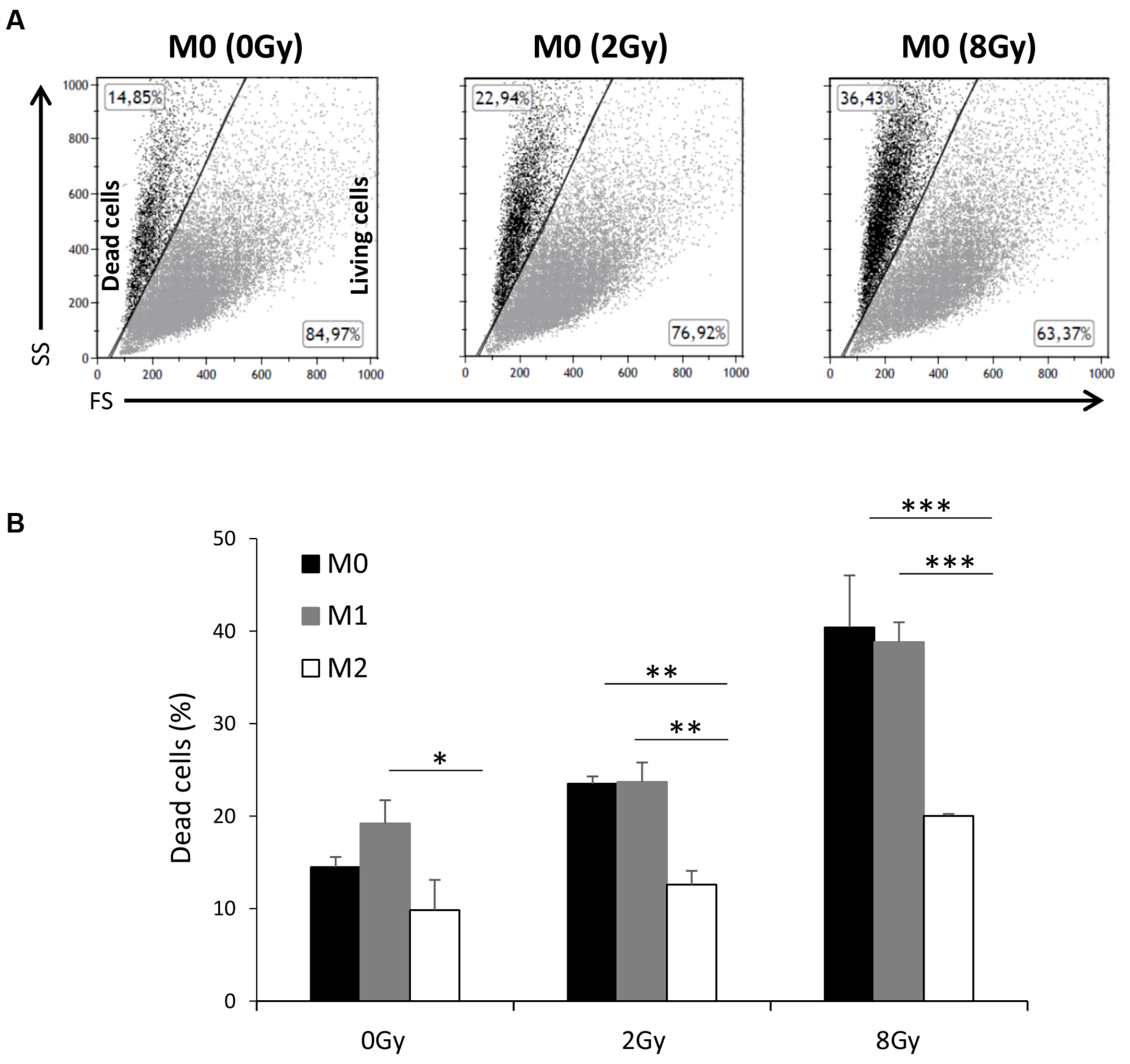

Supplementary Figure 3: MΦ cell debris after irradiation. (**A**) SS (side scatter, the parameter reflecting cell granularity)/FS (forward scatter, the index of cell size) flow cytometry profiles of M0 MΦ 72h after 0Gy, 2Gy and 8Gy radiation. The gates of dead cells and living cells were noted on the control M0 MΦ (0Gy). (**B**) Quantification of dead cells from the proportion of cell debris in M0, M1 and M2 MΦ 72h after 0Gy, 2Gy and 8Gy radiation. Mean ± SD, n=3 different experiments per condition. Statistical significance was achieved when $p<0.05$(*), $p<0.01$(**) and $p<0.0001$(***).
